# Gerontology, Art, and Activism: Can the Intersection of Art, Social Research, and Community Power Lead to Lasting Change?

**DOI:** 10.1093/geront/gnad090

**Published:** 2023-07-11

**Authors:** Sarah Campbell, Elaine Dewhurst, Atiha Chaudry, Ruth Edson, Rohina Ghafoor, Marie Greenhalgh, Suzanne Lacy, Tendayi Madzunzu

**Affiliations:** Department of Social Care and Social Work, Manchester Metropolitan University. Manchester, UK; Department of Law, University of Manchester. Manchester, UK; Greater Manchester Black, Asian, Minority Ethnic Network, Manchester, UK; Manchester Art Gallery, Manchester, UK; Manchester Black Minority Ethnic Network, Manchester, UK; Wythenshawe Good Neighbours, Wythenshawe, Manchester, UK; Roski School of Art and Design, University of Southern California, Los Angeles, California, USA; ZIWO, Manchester, UK

**Keywords:** Decolonization, Gender, Intersectionality, Participatory research, Socially engaged art

## Abstract

This paper seeks to address the question of what gerontologists and humanities scholars can learn from how their respective fields engage with critical issues of age-based intersectional disadvantage, inequality, colonialism, and exclusion. The paper considers the Uncertain Futures Project, a participatory arts-led social research study based in Manchester, United Kingdom. The project explores the inequalities of women over 50 regarding issues of work using an intersectional lens. This work has produced a complex entanglement of methodological ideas that underpin performance art, community activism, and gerontological research. The paper will consider if this model can lead to a lasting impact beyond the scope of the project and beyond the individuals involved. First, we outline the work undertaken from the conception of the project. We consider the relationship between these activities and the ongoing nature of qualitative data analysis within the complexity of academic workloads and competing priorities. We raise questions and considerations of how the elements of the work have connected, collaborated, and intertwined. We also explore the challenges within interdisciplinary and collaborative work. Finally, we address the kind of legacy and impact created by work of this nature.

This paper analyses an interdisciplinary arts-led participatory research study in Manchester, United Kingdom, to explore how the different aspects of the project work together to create a coherent piece of work, while acknowledging and unpacking the tensions that emerge throughout the project. In particular, we consider how social scientists, artists, and community activists together successfully address age-based intersectional disadvantage, inequality, colonialism, and exclusion. We conclude that this kind of collaboration has the potential to create strong alliances that do have significance in creating legacy regarding critical research and artmaking.

Uncertain Futures (https://uncertainfuturesproject.co.uk/) is a participatory arts-led research project, which draws together socially engaged art practice, social research, and community activism in an effort to achieve social change. It involves a variety of actors. The research team works within several social science methodologies (Gerontology, Law, and Social Care). The artist, Suzanne Lacy, is a socially engaged feminist artist who has been working for more than four decades to challenge inequality and campaign for social justice. Manchester Art Gallery (MAG) is a city-owned cultural institution that strives for social impact and societal health through a holistic and purposeful artistic program of art and education for all people. Ruth Edson works as a Community Learning Manager at MAG and is the project lead for Uncertain Futures. The advisory group is comprised of 15 (now 14) women leaders, activists, citizens, and workers from across Manchester who bring not only their own experiences of being women over 50, but also represent the voices of women from their communities. This paper begins by analyzing the inspiration behind and the formulation of the Uncertain Futures project, followed by an unpacking of the methodological complexity of this arts-led research initiative, the actors, and their distinct contributions. We critically assess the role of relationships between the actors in the project before analyzing in more depth the challenges presented by these interwoven relationships.

## The Project

Artist, Suzanne Lacy, was engaged by MAG under the leadership of then Director Alistair Hudson to develop a new artwork in 2019 having worked previously with MAG exploring the precarious working lives of domestic workers in Manchester (https://www.suzannelacy.com/cleaning-conditions/). Lacy and Edson together envisioned a new project which would respond to current challenges facing the city particularly those arising from the significant proportion of older adults economically inactive within the city. Greater Manchester has a higher-than-average number of persons between the ages of 50 and 64 out of work than the rest of the United Kingdom (GMCA, 2018). Research also suggests that more women over 50 are now in the workforce, and yet those women who are out of work are in higher numbers than men in the same age group (C4AB, 2020). Women’s work histories mean that due to the link between pensions and work within the formal economy, they are also less likely to leave work with secure financial futures (Grady, 2015). A recent report by the Runnymede Trust and Fawcett Foundation has identified significant inequalities experienced by women of color at all levels within the workplace (Gyimah et al., 2022). There are also few studies that focus on the experience of mid-life women in the workplace (Austen & Ong, 2010), and there is a need for more understanding of the working lives of this age grouping as well as the conditions and experiences of women from diverse backgrounds.

Uncertain Futures encompasses art and research centered around this topic of older women and inequalities regarding work in Manchester. All Lacy’s previous work has sought to raise the voices of marginalized communities and challenge inequalities; her socially engaged art practice has worked alongside people from the communities she wishes to shed light on, and often takes the form of research, workshops, or dialogue to work toward some kind of “output” such as an event or final piece (Simoniti, 2018). Lacy envisaged the Uncertain Futures project as art *and* research, considering these elements of the project not as separate aspects but as a combination of aesthetics, research, and activism (Simoniti, 2018). The title was taken from the work of Gerontologist Professor Sarah Vickerstaff and her study on the extended working lives of older workers (see Uncertain Futures: Managing Late Career Transitions and Extended Working Life project funded by the Economic Social Research Councils (ESRC) [Ref. ES/L002949/1]), who granted permission for the same title to be used for this arts-led research project. The project has sought to embrace the important role of the arts and humanities in bringing innovative methodologies and new theoretical frameworks to the production of knowledge in aging research (Twigg & Martin, 2015). As Kivnick and Pruchno (2011) suggest, “Humanities and arts scholars draw on analytic methods to address complex questions that have no definitive answers” (p. 143). They provide opportunities to challenge dominant narratives of aging and to support activist accounts of aging experiences (Murray et al., 2014). In the case of this project, we have generated diverse narratives of older women whose transcripts are now exhibited as artifacts on the art gallery walls. The gerontological underpinnings of the study have enabled an analysis of the research in the context of social science understandings of age. But it is through the engagement with aesthetics and the arts, that the project has captured the imaginations of the people of Manchester. The work has facilitated conversations around gender and age in ways unlikely for that of a research report or article in its aim to challenge social inequality and create social change (Kivnick & Pruchno, 2011).

The project began pre-coronavirus disease (COVID) with a consultation with various organizations and women’s groups across Manchester to discuss with older women what they felt were the pertinent issues facing aging women in the workplace. This consultation ended in early 2020, just as measures to contain COVID began to emerge. MAG was closed to the public and planned exhibitions were postponed. However, like many organizations, work went online. Despite an initial pause for consideration of what the project could now look like, the intention was to continue onwards taking learning from the consultation into a series of online conversations that were hosted through MAG with Lacy, older women, academics, policy-makers, and other organizations. These online meetings explored the challenges facing older women in both paid and unpaid work. These conversations continued over several months, and ideas regarding the project began to develop with a plan to stay online but eventually to host some kind of in-person event or exhibition. Working with the researchers, in November 2020, the project hosted an ESRC Festival of Social Science event online and invited participants from across Manchester to consider challenges facing older women in the workplace and to discuss policy solutions. The event was used to publicize the intended work and to launch the project and new advisory group (see https://festivalofsocialscience.com/).

The confirmed advisory group represents a diverse group of women from across the city, and the women are from different racial and ethnic backgrounds, their ages ranging from 50 to 70+, and include women who had newly migrated to the city and others who had lived in Manchester all their lives. Throughout this paper, when we refer to the advisory group, we also mean coresearchers. All the women in the advisory group have been, and continue to be, involved in different aspects of the research process that is entwined with artmaking and civic work. Some of the advisory group self-selected to take on greater or lesser roles in different aspects of the work.

In the next phase of the project, virtual meetings were convened which enabled the advisory group to get to know each other and codesign the project together. A “data matrix” was cocreated which identified the key intersectional inequalities in four areas of focus: accessing work, in-work, exiting work, and, pertinently, the impact of COVID (see gallery version of matrix in Supplementary Figure 1). The work was guided over the next 8 months by Lacy and Edson in terms of the aesthetics, with the researchers leading on developing research questions, the research protocol and securing ethical approvals with the support of the advisory group. Ethics was received through Manchester Metropolitan Health, Psychology, and Social Care Research Ethics and Governance Committee in June 2021 (ref: 27942). Decisions were taken at these weekly meetings, and input was taken on board. Inevitably not all decisions were taken in consensus, and this will be discussed within the paper. The advisory group continued to support the development of the research and provided insight through their lived experience and community knowledge and codeveloped ideas for planned activities for an exhibition and civic program when MAG reopened its doors to the public.

In the summer of 2021, the project began Phase 1 of its public-facing exhibition by interviewing 100 women in MAG. The women were interviewed by the researchers, and accompanied, where necessary, by a translator. The 100 participants came from diverse backgrounds, and two thirds were from minority ethnic backgrounds. MAG housed the interview room, and during interviews, a blind was drawn and white noise was played to protect the anonymity of the women (see Supplementary Figure 2—the interview room). The anonymized transcripts were hung on the gallery wall as artifacts, accompanied by a film sharing the voices of the advisory group telling their own stories. In March 2021, we held a celebration dinner that brought together the 100 participants to celebrate their stories which was made into a short film. The exhibition has now moved to Phase 2 which is an evolving exhibition sharing our methodology and findings (see Supplementary Figure 3—current exhibit).

## Methodological Interweaving: Methods and Actors

The work is underpinned by three distinct frameworks: Participatory Research; Socially Engaged Art Practice; and Community Activism, all of which are coaligned by egalitarian principles regarding distributions of power. The various actors in the project play a distinct role in these methodologies.

Lacy, recruited as an “artist,” tends to begin projects with a premise or a challenge, but the nature of the work is often undefined. Her work is influenced by Beuys, Social Sculpture, whose own political and public artwork was pioneering in bringing art into dialog with its audience and public policy and coining the phrase that “everyone is an artist” (Jordan, 2013). Lacy’s work often takes place outside of the “museum” setting and creates public performances that bring people together to codesign projects and outputs around issues that highlight the voices of marginalized groups (Jordan, 2013). Art is used to communicate dissenting ideas, to occupy spaces of protest and to enable the telling of stories that might otherwise not be heard (Jiang et al., 2020). Simoniti (2018) notes that this work is about impact but also method, and that Lacy’s work is significant for its lengthy process of engagement to reach a final piece. Uncertain Futures has been in process for more than 2 years, with Lacy still regularly meeting virtually with the group.

MAG also plays a pivotal role as a proponent of socially engaged practice. Edson, MAG’s Community Learning Manager, works to empower marginalized voices across the city and to engage communities to be a part of MAG’s work. Often this kind of work is off-site or at least not part of public-facing gallery work (Morse, 2021). MAG is a city-owned public building and seeks to be an institution that is “for the people.” This is not without complexity, as galleries are faced with the need to reconsider their roles in the context of postcolonialism (Soares, 2015). MAG is in the process of undertaking this by considering the role of the museum within the city, taking forward the concept of the “useful museum,” and further addressing the role of art in social change (Adams, 2022). The commissioning of Lacy placed socially engaged practice centrally within MAG and signified its changing vision (see Lynch, 2020).

Participatory research is an approach that attempts to reverse the power dynamics in research that is done with, rather than to or on (Kindon et al., 2007), as a “bottoms-up” approach (Cornwall & Jewkes, 1995). It has been variously referred to as participatory action research (Kindon et al., 2007); community-based participatory research (Hacker, 2017); and inclusive research (Higginbottom & Liamputtong, 2015). It has evolved from feminist activism and wider civil rights movements in challenging power in relation to the production of knowledge (Faulkner, 2017; Hacker, 2017). Challenges in participatory research arise with respect to meaningful community engagement (Corrado et al., 2020), especially when operating within the academy or systems of power which continue to operate within hierarchies of knowledge (Faulkner, 2017). Additionally, Corrado et al. (2020) note issues regarding the funding environment that affects researchers as to when participation can begin. Time is also a challenge (Bourke, 2009). We will return to this later.

The advisory group plays a significant role with respect to community activism, particularly as many of them were already successful community activists and disrupters of traditional power structures engaged in community development work. Community activism is focused on engaging in dialog and negotiation within systems and institutions of power (Milligan et al., 2008). These women were invited to be part of the advisory group because they were leaders in their communities. Some women on the advisory group are not community activists per se, but rather they work within anchor organisations and undertake engagement work at a grassroots level, or are women representative of lived experience from the perspective of being a “minority voice”, and through this project are engaging in activism.

All the various actors and methods interwoven in this project are underpinned by a similar approach to engagement: a desire to challenge existing structures of power and to bring marginalized voices to the fore. As a collective, this included being motivated by concerns of inequality and oppression, and violence against communities. Within our weekly meetings, we engaged with the politics of Black Lives Matters (Rickford, 2016) and concerns of violence against women and ageism which were exacerbated during the COVID-19 pandemic (Emmer de Albuquerque Green et al., 2022; Sánchez et al., 2020). Negotiations about power within society and within the project scaffolded our discussions around understanding the impact of inequalities on the lives of women. We argue that the arts and humanities are spaces that allow these kinds of discourse, where there is an understood engagement with difficult and complex questions about societal issues including those concerns that might be less popular or neglected areas of interest (Kivnick & Pruchno, 2011). Cornwall and Jewkes (1995) note that participatory research is more of “an attitude” than a “series of techniques” that are actioned (p. 1671). This is not to say it is easy to achieve equity, even with the good intentions of those involved and within a project focused on challenging inequalities. Perhaps as Rose and Katahil (2019) note “co-production between professionals and service users is fundamentally an unequal relationship despite the promise of a Third Space for collaboration” (p. 8). We can say that even at this stage of the work, we are continuing to discuss the issue of power when it comes to work, such as writing and disseminating our findings or methods of work. We discuss authorship and ownership and what this means to those involved. However, these discussions are vital to encompassing the “attitude” of participation in the project, even if not always perfectly executed.

## Relationship Development and an Ethic of Care

Participatory research and socially engaged practice are essentially about relationships and mutual trust (Groot & Abma, 2019). This project is no different, relationships were central to the work and to making it work.

A great deal of time was spent nurturing relationships and providing care within the project. Initially, the project was developed in an online capacity, where relationships were being built via weekly zoom meetings during the pandemic. These meetings provided a welcome connection for some and a regular routine that arguably supported the development of relationships. Despite this, building relationships online is not an easy undertaking, there are less spontaneous interactions than those that happen face to face (Hall et al., 2021). Some members of the advisory group already knew each other or were connected through their community activism, which undoubtedly helped and contributed to the success and longevity of the project.

However, much of the essential work of nurturing and caring for these relationships occurred outside of these online meetings. Edson, as MAG’s Community Learning manager, was the key facilitator and coordinator for the work. This stability was significant as she was able to invest time in building relationships and respond to any concerns, doubts, and questions. Essentially, Edson was performing “care in the museum” (Morse, 2021). Care is considered here, not simply as something that is done, but it is a “disposition” and this disposition was the thread running through the project (Morse, 2021, p. 204). Certainly, much of the work of the project and the advisory group were concerned with issues of care—“caring” about inequalities, “care” as the work of women, and the inequalities associated with this role for older women (Cancian & Oliker, 2000). “Care” is part of the language of the project and ensuring that the advisory group women felt valued within the project was important in establishing trust. The advisory group has often spoken about this development of trust when asked about the strength of the project.

Some of the women were experienced as stakeholders in previous projects and leaders in their communities and had high expectations for what coproduction should be. Therefore, attention to relationship building was crucial. A pivotal point in the relationship development was the trialing of pilot interviews with the advisory group members in April 2021. This was the first time that members of the group had met face to face, and with the researcher. MAG was still closed to the public, but restrictions were such that under particular conditions people could meet again for work purposes. We arranged to record the women’s own stories relating to their relationship with work for the creation of an exhibit for MAG and to combine this with piloting the codesigned interview questions. It was an emotional day coming face to face for the first time. It felt like a significant moment in the life of the project and the women reflect on these interviews often when discussing the project. Not least perhaps because their voices have since become an important aspect of the gallery exhibition. Perhaps emphasizing the significance of COVID-19, and its impact on all our lives, while being able to be in person again, felt extraordinarily powerful and enhanced those feelings of peer support and care.

The ethic of care that underpinned the project meant that when the advisory group members were tasked with recruiting participants to take part in the data collection aspect of the study, they were able to go to their networks for recruitment with integrity because they could trust that participants would also be cared for. Data collection was undertaken with huge concern for care. The interview room in MAG was an aesthetically pleasing space, with comfortable chairs and a standard lamp. When the doors to the room were closed, and the blinds were drawn, it was a dimly lit and calming space. Many of the women who were attending interviews had never been to MAG before, and we arranged for them to have taxis, if required, to bring them to the gallery. Many of the women attending were accompanied by the advisory group member who had recruited them and they were met by volunteers employed specifically for this role. Many of these volunteers were older women who also took part as participants in the research. The women were escorted to the interview room, and after each interview, the woman was taken to the café for (complimentary) refreshments. A few days after each interview, women were contacted by their advisory group contact or by the Community Learning Manager to check that all was well after the interview. Some women were also contacted at a later point to check the anonymization of their transcripts if they had requested this. These processes were written into the research protocol to gain ethical approval and were integral to how the work was conducted. This kind of care contributed to how many women later choose to attend the 100 women celebration dinner in March 2022 and choose to be filmed, which is now exhibited in Phase 2 of the exhibition.

Morse (2021) suggests that there are a few ways that museums become spaces of social care through their practice and this project is attending to many of them, but particularly the following:

Third, it is a more ‘generous’ way of working, which pays attention to the aims and agendas of individuals and communities. It is more open and transparent about the museum’s own objectives to find collaborative ways forward. Fourth, it addresses issues of power by focusing on bespoke programmes based on person-centred and collaborative approaches where the museum is more directly useful to communities and support organisations (p. 208).

## Challenges to Cocreation: Methods, Actors, and Relationships

Although this project has been a very successful one, it was not without its challenges, some of which we have alluded to above. These hinge mainly on institutional challenges and issues of decolonization. However, we also want to ensure our discussion focuses on solutions and the import of flexibility in arriving at these solutions: Flexibility is key to the success of the project. Although we may not have overcome all these hurdles, and we still have a myriad of questions unanswered, we did manage to resolve some tensions through the use of a flexible approach.

### Institutional Challenges

The Universities as institutions have been incredibly supportive of the project. The drive to social responsibility and social impact by universities is an increasing trend (Barrena-Martínez et al., 2019) and this project meets many of these social responsibility aims. However, despite this positivity, challenges arising from the structural and institutional design and vision of the Universities have emerged. The biggest challenge relates to ethical constraints and funding, and research impact difficulties.

The main difficulty arose in aligning ethical approval with the timeframe of the aesthetic production of the art and the live interviews (Mrisho & Essack, 2021). Ensuring that ethical approval was successfully achieved in time and in a manner appropriate to the artwork; given the protracted timeframes involved in writing up the ethics application, submitting, gaining approval, and making necessary changes to satisfy the ethics board; created a huge amount of tension and stress within the team. Although flexibility is not generally a word associated with gaining ethical approval, it was a key resource in overcoming the tensions in this participatory project. The researchers took on the task of bringing the other actors into the ethical process: Members of the advisory group, MAG, and the artist were intimately involved in the drafting of the ethics application and in understanding the importance of various elements such as anonymity and informed consent, were kept abreast of submission dates and timeframes regarding ethics committee meetings and when approval could be expected.

Naturally, given the nature of the research, the requirement of anonymity was also significant, as was the need for informed consent. The main challenges in achieving this were working out how anonymity was going to be achieved when the main focus of the artwork was the words of these women and a live exhibition, where women would be interviewed in a public space, and how informed consent was going to be obtained from a disparate group of women with varying levels of education, social mobility, and language abilities. Working with all the actors was key to overcoming these challenges. Explaining our concerns as researchers and our needs as part of the ethical process, the group as a whole was able to come up with flexible solutions which ensured these important elements of the ethical approval were met. Many members of the advisory group had previously worked on projects requiring ethical approval and had first-hand knowledge of what was required and how it could be achieved in such circumstances. In addition, as the advisory group was involved in recruiting the women for the project, they also had many suggestions on how best to ensure informed consent in the cohort of women they were responsible for.

The first, anonymity, was achieved through only placing anonymized interviews on display in MAG, and developing an agreed anonymization protocol, and the second, informed consent, through carrying out the consent process in advance through information provision and at the start of the interview after a short discussion between the interviewer and the interviewee (including their translator). Using the team of actors in this way was pragmatic and flexible but also a beneficial process bringing the group closer together in terms of the development of the project and creating a better understanding of the research process and its timeframes. It also helped to create more achievable research expectations in the longer term. With regard to the use of MAG as an interview space, the original design of the interview booth had to be adjusted. Doors were fitted that could be closed when an interview was in process, the viewing window had a blind fitted, and after testing sound within the booth for anonymity, MAG technicians helped to develop a solution using a white noise machine to play when interviews were taking place. It took time to negotiate the volume at which this could be played to ensure anonymity, while also not causing disruption to other staff and visitors in MAG. However, it did also act as a useful signal that something was happening in the interview booth. These negotiations were vital for the research team who understood the importance of anonymity in obtaining ethical approvals in very different terms to some other members of the project.

Further, and interrelated, challenges arose from an institutional focus on the research excellence framework and funding pressures (Gray et al., 2000). These challenges are not exclusive to our project and have been well documented in academic literature (Hughes, 2016). In terms of developing an impact case study (which is a natural fit for this project), the main challenges arose from identifying opportunities for, and evidencing, impact. This was hampered by a lack of funding which meant that much of this work had to be done in the allocated research time of the researchers, with concomitant impacts on other research projects, the timeframe of the project, and the ability to develop certain aspects of the project more fully. To mitigate these challenges, the team decided to be flexible with respect to the outputs of the project and took opportunities to “write” impact into various activities occurring alongside the main aesthetic productions involving relevant stakeholders at various stages of the project who may have an impact on policy development. It was also important to take time to evidence research impact without undermining the forward nature of the project by asking people to reflect on their actions so far. We achieved this through a “postcard pledge” at a policy event and by following up with all participants at the 100 women dinner. Still, difficulties remain: Evidencing and developing impact plans can be difficult in the absence of funding to ensure the coherency of the project and to allow time for research paper development. We aimed to be as flexible as possible with deadlines and funding applications but the struggles still remain.

### Decolonization Challenges

The research team is a tight-knit one: It comprises mainly two social science researchers from different backgrounds, supplemented at various times by research assistants who have been integral to the development of various parts of the project. It also includes four key members of the advisory group. Some of these women have research-related experience, but this is not a necessary condition for being on the research team. The main condition is interest in the research and some experience (either personal or professional) in the issues subject to analysis. The team has been extraordinarily cohesive from the outset with similar outlooks, goals, and aspirations, and each team member bringing something different and worthwhile to the analysis.

One issue that concerned the University-based researchers from the outset was one of “whiteness” (Stam, 2021) and the impact of this on the research in terms of defining material realities, discourses, and ideologies (Cole, 2020; Wekker, 2016). As the project developed so dynamically, the methodologies were not developed with this issue in mind—rather the methods were to some extent predetermined by the artist and MAG, and the researchers developed the methods around this and all were negotiated with the advisory group. This has meant that efforts to incorporate an ethic of solidarity in the research have been rather ad hoc. Stam (2021) has indicated that there are five key components in activating solidarity. A reflection on these activation constituents indicates some of the power of participatory research in achieving solidarity, as well as the greater efforts needed, in our project at least, to fully address privilege or lack of it.

As a first step, Stam (2021) suggests that white researchers should become intimately connected with their privilege, especially those attributes which afford us “existential luck” (Vest, 2013). In conducting her participatory research, Stam (2021) engaged in debriefing sessions, storytelling, and sharing. What Stam (2021) is essentially advocating is reflexivity: an awareness of one’s own position in the research. However, care must be taken that such actions are not performative (Han, 2023). In our research, we tried to move beyond the performative to develop relationships and opportunities for sharing and debriefing through weekly conversations with the Advisory Group, asking for feedback (however negative), and building the project through the suggestions of the women themselves. The relationships that were developed were deep, particularly between the advisory group women, who have described important life events happening during the project, including bereavement and illness, which other group members have supported them through. Although it is fair to say that the researchers, artist, and community learning manager have shared less personal stories, over time it has been inevitable that personal lives are shared to some extent. This has happened partly through Zoom conversations that are held in our homes with children or pets becoming part of the meetings. This helps to blur boundaries and create solidarities. COVID-19, rather paradoxically, did enable us to spend time online probing our positionality, thinking more reflexively, and activating solidarity in the project. This has certainly been reflected positively by the advisory group.

Stam (2021) also advocates interrogating epistemic assumptions and ensuring that research is disseminated in a way that allows others to critically engage in the research. As social scientists, we have to take particular care to challenge existing knowledge because we are often, as Han (2023) identifies, programmed to ensure that our research builds on existing frameworks that may have colonialist underpinnings. This was one of the key elements of the project which was considered thoroughly from the outset and the artist was rigorous in ensuring that the cohort of women interviewed and those on the advisory group represented the diversity of Manchester. The progress of the project which has created “teams” to work on various aspects of the project from workshops to research outputs has been carefully developed to ensure that all voices are heard. However, there are some key issues we should have addressed differently, especially around removing leadership (or at least perceived leadership) roles which would have reduced potential assumptions and redistributed power in a more equitable way. This speaks strongly to the challenge of accommodating or supporting research that potentially objectifies racialized people (Han, 2023). We must, as the project progresses, work harder to ensure that the processes, methods, and knowledge we use actively avoid condoning such objectification (Jack & Westwood, 2009).

This latter point also links to another key component of activating solidarity: that of disrupting hierarchies of power (Stam, 2021). Participatory research is particularly well suited to privileging the “expertise of lived experience, self-monitoring power, foregrounding relationships between bodies, ideas and institutions and catalysing change and action” (p. 191). By making the process participatory, the project aimed to shift the balance away from the traditional researcher dynamic and hand more power and direction to the participants. Drawing on educational research around decolonization of the curriculum, the crucial focus for us was on “reversing or exchanging the positions of the oppressor and oppressed,” rather it is about “dismantling those power structures, or at the very least undermining them to begin with” (Mendes & Lau, 2022, p. 233). To achieve this dismantling exercise, the team worked in a strongly coproductive way. The advisory group was instrumental in codetermining the research questions, the research matrix guiding the research agenda, determining recommendations, developing a civic program of events to run alongside the exhibition, including a partnership with Manchester City Council Work and Skills team through which seven events were held that directly addressed issues raised in the interviews, and disseminating many of the research outputs. The advisory group alone developed their own story-sharing event where they re-enacted the interviews and had full directorial control over the content and methods of sharing. However, as mentioned above, leadership roles are attributed to researchers, coordinators, and artists, in various measures, which still allows certain power dynamics to play out. Within participatory projects, there is often a driving force, and it is not without challenge to ensure that there is equality within groups in terms of decision-making (Groot & Abma, 2019). Within Uncertain Futures, decision-making did happen outside of group meetings at times, to move the project forward by those employed—paid to do so—and responding to pressures from employers/timescales (Groot & Abma, 2019). As Stam (2021) also experienced, this is often caused by institutional expectations (such as financial pressures and research expectations) which therefore require leadership roles to be played out more rigidly. Overcoming this is a difficult challenge that we did not, unfortunately, find answers to, although our more flexible approach to leadership at times did assist in dampening some of the more damaging effects of this on the project overall.

In any research project, it is important that all parties pay attention, listen, and take action when needed (Stam, 2021). When attempting to activate solidarity, this is even more important to ensure that asymmetrical dialogs are shifted. This can require “opening up space for fraught, tense conversations, and not avoiding or shutting down critical space even when accompanied by negative affect” (p. 191). On reflection, this is perhaps the weakest area of our project. We have until rather recently, when the research aspect of the project began, failed to have these types of discussions. We did not move beyond bipolarity (Mendes & Lau, 2022) and we resisted (by omission) a connection with the voices within the pluriverse (Mignolo, 1995). As we approach the next phase of the project and begin the process of these conversations, we have to admit that this is something that would certainly have been more beneficial earlier on in the project. It would perhaps have opened up a greater opportunity for honesty and reduced reliance on leadership roles which may ultimately have hampered the development of the project and its outputs.

Finally, Stam (2021) indicates that it is important, particularly in this phase of the project, to practice decolonization research by “attending to the historical and current context of colonialism our research operates within, and exploring alternative models of knowledge production, ‘ownership’, and dissemination” (p. 193). If we fail to do this, we will effectively be allowing coloniality and white academic privilege to absent alternative voices (Han, 2023). We have been mindful of engaging the advisory group in the determination of research questions. Their personal inputs have been invaluable in identifying and devising research and dissemination strategies and recommendations. As Stam (2021) indicates, however, this is a massive time investment and often clashes with the rigors of ethics review committees, university expectations, and funding timeframes. However, in this project, this has perhaps been one of the strongest aspects of our work: Ensuring voice to the participants to make this research their own and to provide a safe space in MAG for the participants, the Advisory Group, and the wider public to reflect on how social change can be implemented and how social inclusion can be achieved. However, this does not address the central power dynamics in our research disciplines: something we must remain alert to, cautious of, and work to overcome.

## Conclusion and Legacy

Conclusion feels like a very final term while working on an evolving project. However, even at this point, we feel confident in concluding that collaborative and participatory research, particularly between community organizations, academia, and public spaces, like MAG, can successfully contribute to interdisciplinary research, particularly in the context of aging where there is often very little attempt to engage in meaningful participation and engagement. The value in bringing together critical gerontological research with the humanities and arts brings a richer and arguably more powerful platform from which to argue for social change. Through the ability to bring alternative depictions of diverse ageing experiences which illuminated the voices of marginalized older women using an engagement with aesthetics, Uncertain Futures has at its core a call to action (Jiang et al., 2020). Additionally, we can attribute much of the success of the project to its participatory processes such as the codevelopment of the research questions which have been clear and forward focused from the outset. Flexibility has also been our friend, whether it has been our ability to adapt to online form or the ability to reassess our research questions as situations developed (such as the impact of COVID-19 on the participants) and we would encourage other research teams to remain flexible in participatory research as it can be important to adapt to changing circumstances. Another feature of our success has been the stability of the project team, in terms of researchers, MAG staff, the Advisory group, and the artist. As discussed earlier, relationships have been key to ensuring the project has the impact we desired but this stability and cohesiveness requires work. In our case, Edson at MAG was the “glue” which held the entire project together.

However, we have not completed this project without challenges, of which the most difficult have been institutional and decolonization. Remaining flexible and coworking as much as possible with the women involved, we have been able to overcome some of the ethical and research issues we have encountered. The building of strong relationships through cocreation has been key to our success in ensuring the research is decolonized and that certain power dynamics are addressed. However, there is more that we could achieve if we had focused our attention on plurality of voice and as we move forward with the project our central concern will be on ensuring that the research focuses on absent voices so that these can be heard. We would encourage other researchers to address such issues at the project outset as the most effective way of achieving decolonization and the many associated benefits.

Turning to the question of legacy, we feel that this is the most enduring and exciting aspect of this participatory research project. Naturally, we had set out to create some intended outcomes such as the public art exhibition, a website, aesthetic productions, funding applications, and research papers. However, many of our greatest achievements have been unintentional or, at least, were reactive to events happening around us, for example, a submission to the Pensions Commission on the change in pension age. The active nature of the advisory group women means that they have also taken aspects of the project and created a series of public events to accompany the art exhibition and have brought ideas back into their own communities to create spin-off productions. Vella et al. (2021) note that legacy in the arts can also be intangible, and our experience has certainly been similar (p. 106). Participants have spoken about the increase in their self-confidence from sharing their stories, the importance of the project from the perspective of social inclusion by bringing women to MAG who might not otherwise have entered such spaces, and the sharing of stories which has the power to, as Vella et al. (2021) so eloquently put it, “spread information on a scale that is usually broader than the community within which the project was carried out” (p. 106). We hope this is the enduring legacy of the Uncertain Futures Project. Winning the Manchester Culture Award 2022 for the promotion of Equality and Social Justice is a recognition that this project has already improved the lives of women in Manchester but also that it has the potential to improve the lives of their daughters and granddaughters for many years to come.

## Supplementary Material

gnad090_suppl_Supplementary_Material

## Data Availability

Data and Research Materials are not currently available because authors have not completed their original work with the data set. This study is not pre-registered.
