# Supplementary material for: Gerontology, Art, and Activism: Can the Intersection of Art, Social Research, and Community Power Lead to Lasting Change?
Source: Gerontologist. 2023 Jul 11;63(10):1654–62. doi: 10.1093/geront/gnad090 (PMC10724043; doi:10.1093/geront/gnad090)

**Online Supplementary Material**

Supplementary Figure 1. Data Matrix. Design Stephen Caton. Photograph Michael Pollard.


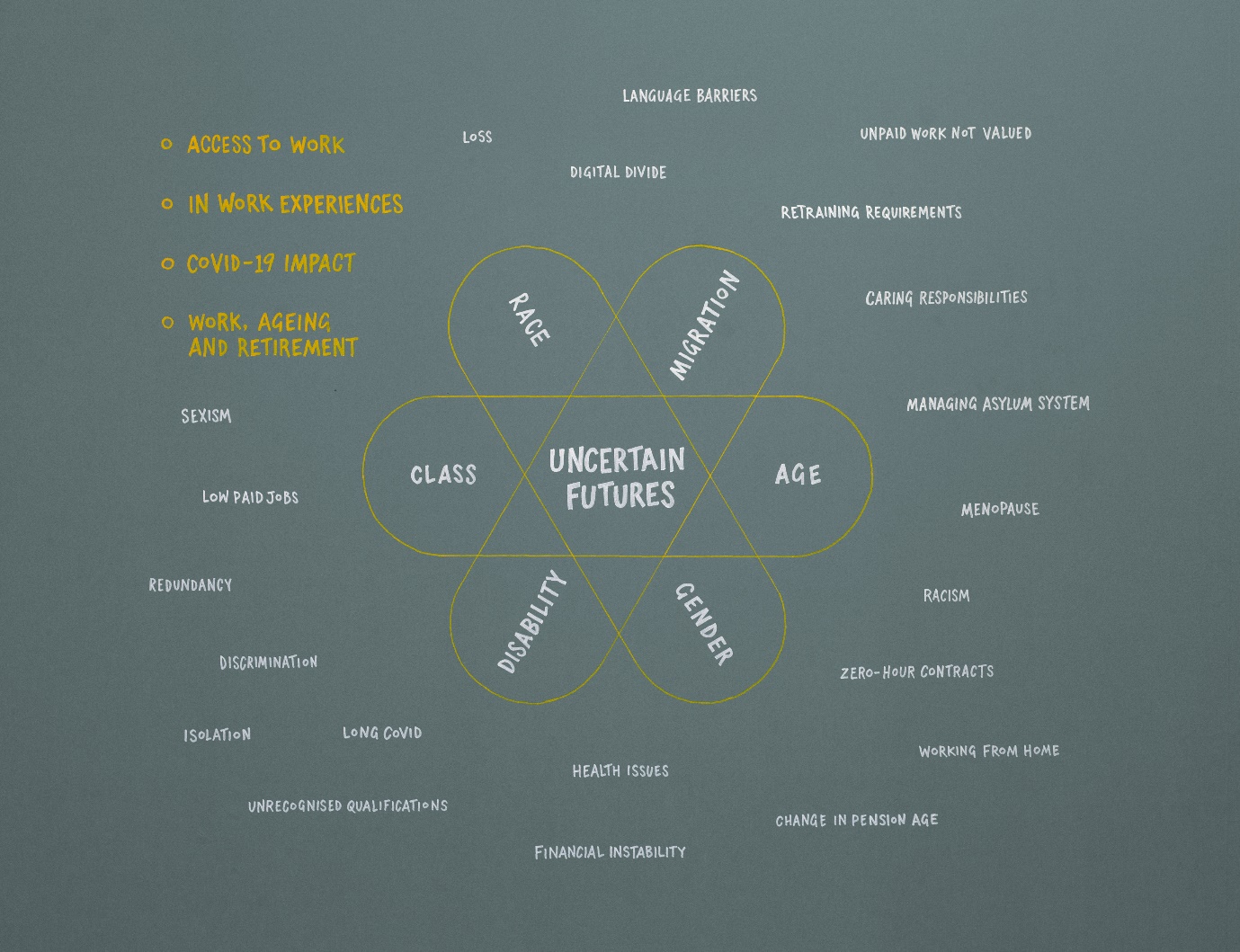


Supplementary Figure 2. Mock interview in process. Photograph Andrew Brooks


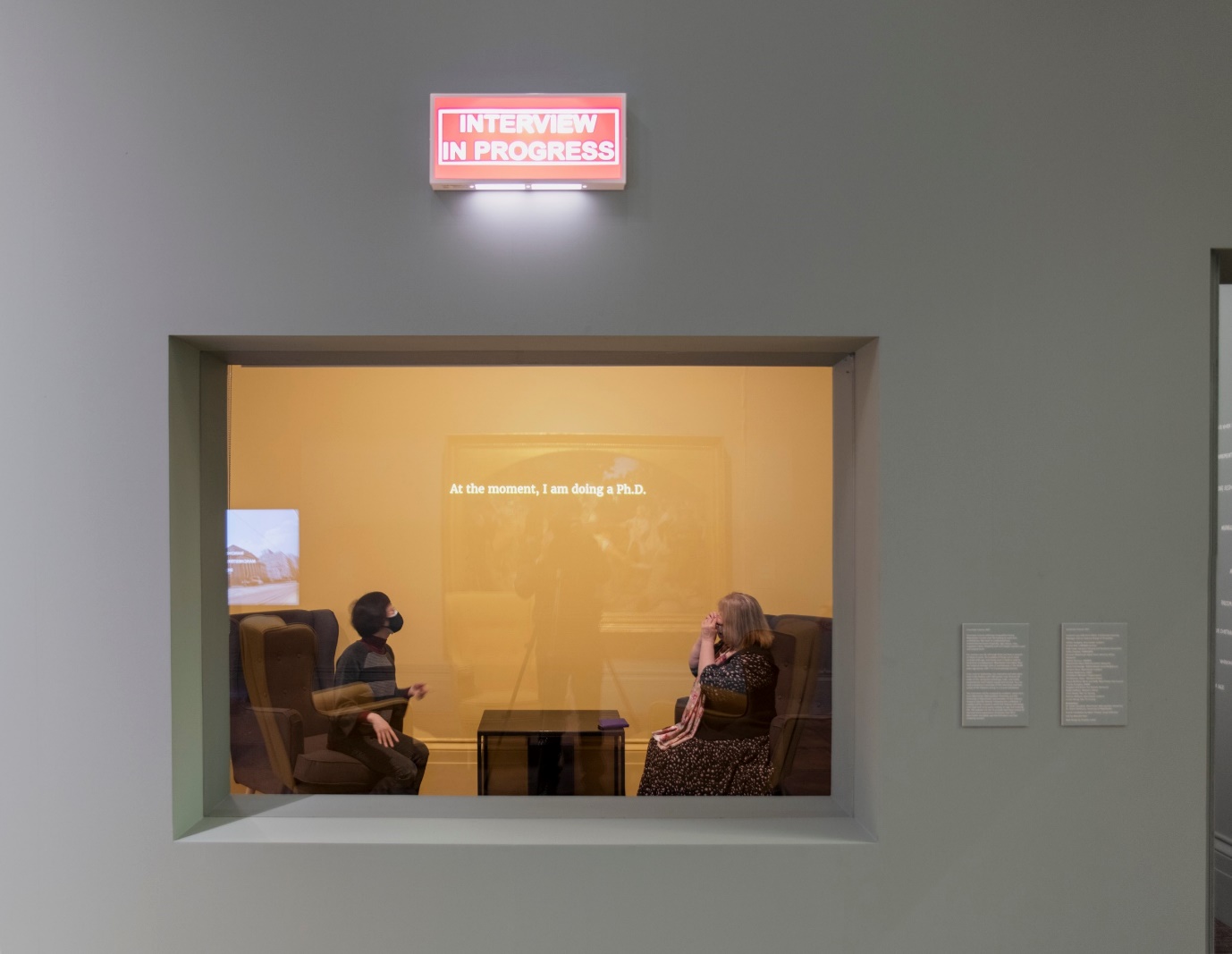


Supplementary Figure 3. Exhibition Phase 2. Photograph Michael Pollard.


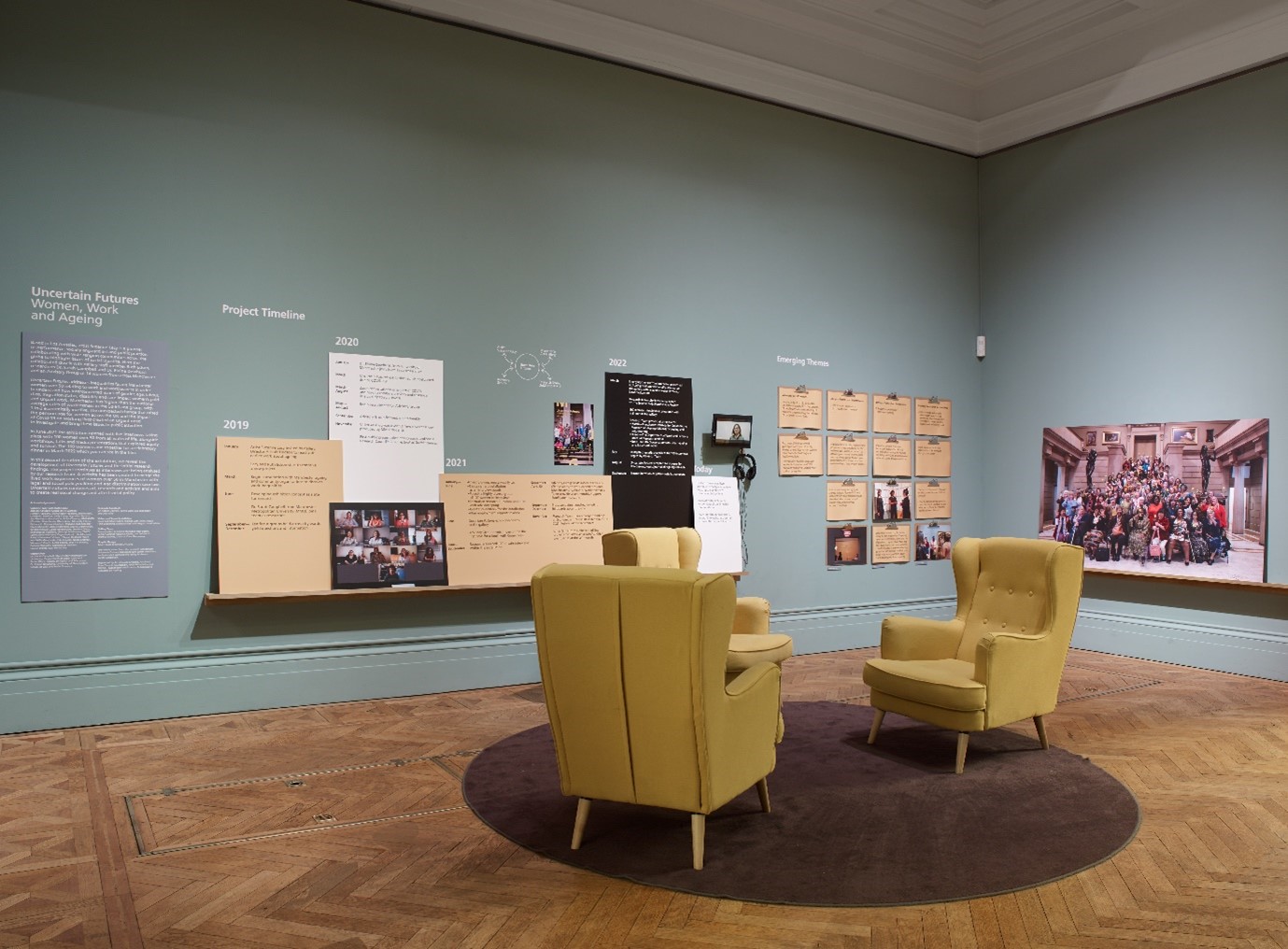

Supplement: gnad090_suppl_Supplementary_Material [file gnad090_suppl_supplementary_material.docx]
